# Individualized closed-loop TMS synchronized with exoskeleton for modulation of cortical-excitability in patients with stroke: a proof-of-concept study

**DOI:** 10.3389/fnins.2023.1116273

**Published:** 2023-05-25

**Authors:** Neha Singh, Megha Saini, Nand Kumar, M. V. Padma Srivastava, Amit Mehndiratta

**Affiliations:** ^1^Centre for Biomedical Engineering, Indian Institute of Technology, New Delhi, India; ^2^Department of Psychiatry, All India Institute of Medical Sciences (AIIMS), New Delhi, India; ^3^Department of Neurology, AIIMS, New Delhi, India; ^4^Department of Biomedical Engineering, AIIMS, New Delhi, India

**Keywords:** stroke, transcranial magnetic stimulation, exoskeleton, closed-loop brain stimulation, neuro-rehabilitation, motor evoked potential, spasticity

## Abstract

**Background:**

Repetitive TMS is used in stroke rehabilitation with predefined passive low and high-frequency stimulation. Brain State-Dependent Stimulation (BSDS)/Activity-Dependent Stimulation (ADS) using bio-signal has been observed to strengthen synaptic connections. Without the personalization of brain-stimulation protocols, we risk a one-size-fits-all approach.

**Methods:**

We attempted to close the ADS loop via intrinsic-proprioceptive (via exoskeleton-movement) and extrinsic-visual-feedback to the brain. We developed a patient-specific brain stimulation platform with a two-way feedback system, to synchronize single-pulse TMS with exoskeleton along with adaptive performance visual feedback, in real-time, for a focused neurorehabilitation strategy to voluntarily engage the patient in the brain stimulation process.

**Results:**

The novel TMS Synchronized Exoskeleton Feedback (TSEF) platform, controlled by the patient’s residual Electromyogram, simultaneously triggered exoskeleton movement and single-pulse TMS, once in 10 s, implying 0.1 Hz frequency. The TSEF platform was tested for a demonstration on three patients (*n* = 3) with different spasticity on the Modified Ashworth Scale (MAS = 1, 1+, 2) for one session each. Three patients completed their session in their own timing; patients with (more) spasticity tend to take (more) inter-trial intervals. A proof-of-concept study on two groups—TSEF-group and a physiotherapy control-group was performed for 45 min/day for 20-sessions. Dose-matched Physiotherapy was given to control-group. Post 20 sessions, an increase in ipsilesional cortical-excitability was observed; Motor Evoked Potential increased by ~48.5 μV at a decreased Resting Motor Threshold by ~15.6%, with improvement in clinical scales relevant to the Fugl-Mayer Wrist/Hand joint (involved in training) by 2.6 units, an effect not found in control-group. This strategy could voluntarily engage the patient.

**Conclusion:**

A brain stimulation platform with a real-time two-way feedback system was developed to voluntarily engage the patients during the brain stimulation process and a proof-of-concept study on three patients indicates clinical gains with increased cortical excitability, an effect not observed in the control-group; and the encouraging results nudge for further investigations on a larger cohort.

## 1. Introduction

Repetitive-Transcranial Magnetic Stimulation (rTMS) is a potential therapeutic modality post-stroke that can facilitate neuroplasticity through the process of restoration of transcallosal interhemispheric inhibition by targeting Long-Term-Potentiation (LTP) and Long-Term-Depression (LTD) through high-frequency stimulation of the ipsilesional and low-frequency stimulation of contralesional-hemisphere, respectively (Hoogendam et al., 2010; Thabit et al., 2010; Edwardson et al., 2013, 2014; Rossini et al., 2015). However, these rTMS frequencies are predefined (such as 1/5/10 Hz) and involve a non-specific, passive-stimulation process where the patient lies in the TMS chair comfortably with the TMS coil on the head. Since, the patient is not voluntarily involved in the brain-stimulation protocol, at times; the patient is not responsive to treatment or might even be asleep during the sessions. The predefined frequencies used are independent of the current brain-state of the patient and do not engage the patient voluntarily or directly in the brain stimulation protocol (Edwardson et al., 2013). The whole passive-stimulation process lacks feedback to the patient, hence, is referred to as an open-loop therapeutic approach. Various factors being the determinant of the therapeutic outcomes apart from brain stimulation parameters (such as phase, frequency, and intensity), its effect is also highly dependent on the current-states of the brain, and without personalization of brain-stimulation protocols, we assume an one-size-fits-all approach (Mitchell et al., 2007; Edwardson et al., 2013).

Unlike these open-loop approaches, Brain-State-Dependent-Stimulation (BSDS) or Activity-Dependent Brain Stimulation (ADS) paradigm is an alternative that employs a closed-loop approach to facilitate focused neuroplasticity. Neuroplastic changes presumably occur in connections between motor cortical neurons firing naturally during the generation of voluntary muscle-contraction and those artificially stimulated by brain stimulation, a mechanism of hebbian plasticity (Gerstner, 2011; Edwardson et al., 2013, 2014). LTP can also be induced by pairing protocols, e.g., associative or hebbian LTP, which allows activity-dependent modification of synaptic-strength by synchronous activation of neurons which is the basis of learning and memory (Martin et al., 2000; Thabit et al., 2010; Edwardson et al., 2013). ADS entails making brain stimulation contingent on voluntary neural/muscle activity and hence, the brain is stimulated depending on its current state using the bio-signal (neural or muscle-activity), as opposed to a fixed frequency in an open-loop approach in rTMS, providing a method of invoking Hebbian mechanism by pairing each episode of motor-activity, and brain stimulation (Edwardson et al., 2013, 2014; Gharabaghi et al., 2014; Kraus et al., 2016; Mrachacz-Kersting et al., 2019).

Different cellular investigations evidenced that the connections between the two neurons are strengthened when the firing of one neuron repeatedly contributes to the firing of another neuron (Bi and Poo, 1998, 2001; Feldman, 2000). A similar effect was demonstrated with stimulation of the median nerve at the wrist paired with TMS, in healthy subjects, which can lead to LTP or LTD-like effects, depending on their relative timing (Jung and Ziemann, 2009). Compelling evidence exists in favor of the ADS paradigm documenting potentiating effects and consistent strengthening of specific connections between neurons in the motor cortex in animals such as primates (Jackson et al., 2006) and rodents-models (Rebesco et al., 2010; Edwardson et al., 2013). Primates’ animal studies evidenced that triggering motor cortex stimulation from contralateral muscle-activity produces neuroplasticity effects. Activity-dependent single-pulse TMS on healthy subjects (Bütefisch et al., 2004; Thabit et al., 2010; Edwardson et al., 2014), and even in stroke survivors (Bütefisch et al., 2004; Izumi et al., 2008; Buetefisch et al., 2011) has also been started in the last 2 decades, evidencing induced motor learning (Bütefisch et al., 2004), increased cortical-excitability (Thabit et al., 2010), and subtle evidence of neuroplasticity (Buetefisch et al., 2011). These rare seminal studies indicated the feasibility of Activity-Dependent single-pulse TMS, where motor activity from affected-hand triggers TMS to the lesioned motor cortex. These studies also indicate the importance of ADS in designing protocols to capitalize on the unique physiology resulting in robust neuroplasticity (Edwardson et al., 2013).

These studies used stimulation in strict temporal relation with the movement attempted which showed increased cortical-excitability, demonstrating the ability of Electroencephalogram (EEG)/Electromyogram (EMG) signal stimulating the brain to drive the cortical-plasticity with LTP-like effects (Bütefisch et al., 2004; Thabit et al., 2010; Edwardson et al., 2013, 2014; Schaworonkow et al., 2018). The studies on stroke-survivors (for one or more sessions) are present in which stimulation is used in strict temporal relation with the movement attempted (Izumi et al., 2008; Buetefisch et al., 2011; Walter et al., 2012; Gharabaghi et al., 2014; Kraus et al., 2016; Mrachacz-Kersting et al., 2019; Wu et al., 2019). However, these studies were “ADS” only in terms of using their bio-signal for triggering brain stimulation. Very few studies have attempted to close the loop in ADS “via feedback,” a crucial phenomenon in stroke rehabilitation (Buetefisch et al., 2011; Gomez-Rodriguez et al., 2011; Gharabaghi et al., 2014). In addition, out of these studies showing the effect of closed-loop ADS in healthy subjects (Izumi et al., 2008) as well as chronic stroke-population, over one or more sessions, only few studies have evaluated the therapeutic-effectiveness of Activity Dependent TMS on stroke patients (Buetefisch et al., 2011; Revill et al., 2020). Even though ADS studies are documented in literature, its effect with various types of feedback is not explored. Moreover, ADS can encourage impairment oriented functional-plasticity by focusing on the impaired and functionally important muscle such as Extensor Digitorum Communis (EDC), by stimulating its respective cortical-representation, for a focused rehabilitation strategy unlike stimulating Abductor Pollicis Brevis (APB) muscle used commonly (Thabit et al., 2010; Edwardson et al., 2013).

Spasticity and Flexor-Hypertonia (FH), which is one of the most common symptoms of stroke, leads to impaired Activities of daily-living (ADL). If not rehabilitated, may cause deformities, and even contractures and can further hinder any therapeutic intervention. Reducing the spasticity of muscles is the initial step in stroke rehabilitation followed by the ADL-training (Singh et al., 2019a). If a patient with spasticity has to be involved in a therapy that is meant to be voluntary and with movement, therapy should take the spasticity as a critical consideration. Till now, no brain stimulation study has considered the spasticity factor in the intervention protocol or evaluated the therapeutic effectiveness of BSDS focusing on spasticity. Spasticity is an important challenge and rarely discussed obstacle in stroke-rehabilitation literature, which might be pertaining to the challenges involved in dealing with spasticity in patients with stroke.

For making the brain stimulation contingent on voluntary muscle-activity, we designed an Activity-dependent TMS system with a two-way feedback novel protocol individualized for patients with different spasticity. We attempted to close the ADS-loop via feedback to the brain through proprioceptive and visual feedback, to voluntarily involve the patient throughout the process. Our hypothesis was if providing brain stimulation while the motor cortex is engaged in generating movement, providing proprioceptive feedback (via exoskeleton-device; Singh et al., 2019a) to the brain by assisting the voluntary-attempted movement, along with visual performance-feedback, in real-time, could potentially improve post-stroke motor-recovery. For motor training, an exoskeleton device (Singh et al., 2019a) was used which can assist the patient in completing the movement, giving proprioceptive feedback to the brain. Considering the criticality of spasticity and feedback in stroke-rehabilitation, and most importantly individualization of the TMS protocols, our goal was to design and develop a novel customized brain-stimulation platform that can be patient-specific according to their clinical presentation, establish the patient-specific novel protocol, perform demonstration study, and at last carry out the feasibility-study for patients with stroke, WRT control group for 20-sessions and take the subjective-feedback of patients.

## 2. Materials and methods

For an Activity-Dependent TMS system with a feedback approach, the fundamental assumptions of system design are: (i) the patient needs to be directly and actively engaged during the brain stimulation protocol to generate voluntary bio-signal and (ii) TMS must be induced/ triggered by bio-signal generating the voluntary movement. This intention of the voluntary movement (is detected in the bio-signal) was synchronized with TMS, making the process of brain stimulation dependent on the current state of the brain. To entail the patient voluntarily in the therapeutic intervention, an exoskeleton device (Singh et al., 2019a) was used to assist to complete the movement attempted as and when intended by the patient. IRB approved the study (protocol-number-IEC/NP-99/13.03.2015) and a pilot study was registered (ISRCTN95291802). All patients signed the written informed consent. The study was designed in clinical settings and a clear description of the method and the intervention is presented below.

### 2.1. Aim of the study

The aim of this novel customized platform was that once the intention of the movement is detected through bio-signal, it triggers both the TMS-pulse and the exoskeleton device. Hence, we designed a system pairing each episode of voluntary motor activity with TMS, where the motor activity from the affected hand of the patient triggers TMS to the lesioned motor cortex. The goal of this study was to design and develop a platform with a novel protocol and perform a demonstration study along with a feasibility-study wrt control group.

### 2.2. Materials

#### 2.2.1. Muscle-selection

**Figure 1:**
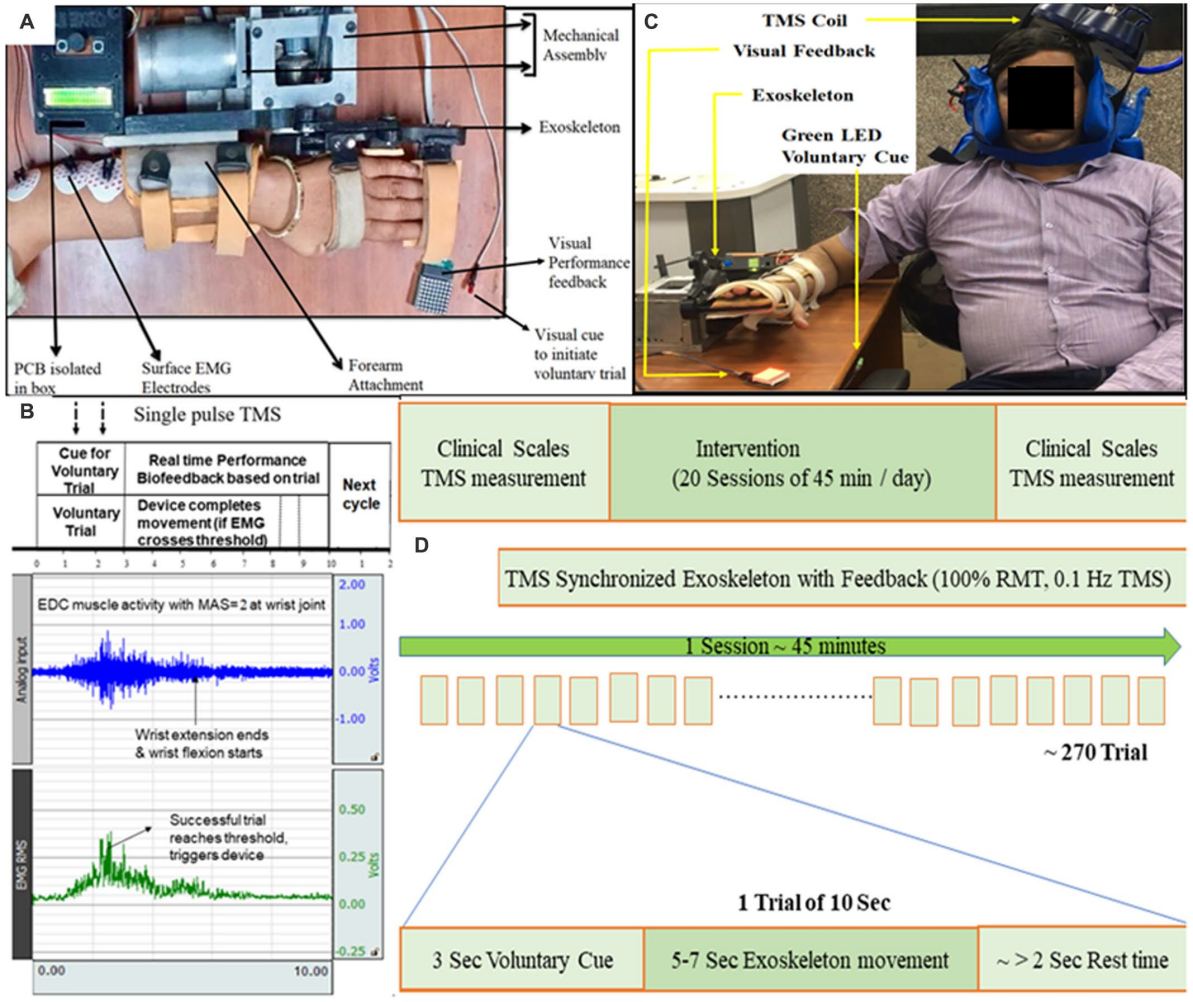
**(A)** Exoskeleton mounted on the hand of a representative patient. Baseline position: wrist in neutral position with fingers extension. Final position: wrist extension with fingers flexion. Motion sequence: Baseline to Final to Baseline position. **(B)** Protocol showing EMG activity during a 10-s trial. 10 s included a “voluntary cue” for attempting the wrist extension (3 s), completing movement (5–7 s), and remaining as the “rest-time” (remaining in 10 s). Raw EMG signal (blue, sampling frequency of 1,000 Hz and gain = 2,000) was filtered (Bandpass-filter = 20–500 Hz, Notch-filter = 50 Hz), amplified and Root Mean Square (RMS; green) was calculated. The patient’s effort detected in EMG simultaneously triggered the exoskeleton device and TMS-pulse; TMS-pulse was delivered within the first half of the EMG burst generated by EDC (within the time of voluntary-trial time) as soon as the predefined activation threshold on RMS-EMG is crossed. If the threshold is not reached by the patient, the 10-s trial starts again. The effort made by the patient (detected in EMG) is directly proportional to the visual feedback (LED Dot Matrix; **C**) Set-up for TMS synchronized with exoskeleton-device, TMS coil being set to TMS hotspot being EDC muscle cortico-representation. **(D)** Study design of TSEF for patients with stroke (*n* = 3).

The Extensor Digitorum Communis (EDC) muscle of affected-hand was chosen (Figure 1A) as this muscle is often impaired in stroke due to flexor-hypertonia leading to spasticity, also because of its critical involvement in ADL for wrist extension, and easily detectable nature of surface-muscle (Singh et al., 2019a). The disposable gel-based wet Ag/AgCl surface-electrodes were used on the belly-tendon configuration; muscle-contraction causing extension of wrist and extension of third-digit of hand was observed for identification of muscle-belly and electrodes-placement. Electrodes were connected to an EMG amplifier (BIOPAC-MP150, Gentech; Figure 1B).

#### 2.2.2. Configuration of EMG threshold

A 20% of Maximum Voluntary Contraction (MVC) of each patient’s residual EMG-activity was considered as predefined activation-threshold. This activation threshold was set on the Root-Mean-Square (RMS) of the EMG signal of the affected hand and represented target muscle activation (Figure 1B). The activation threshold of RMS-EMG was used to simultaneously trigger exoskeleton-device and single-pulse TMS, whenever voluntary effort-induced EMG activity crossed the predefined threshold. Configurability of the residual EMG, activation threshold, RMS amplitude, and RMS EMG threshold was customized individually for affected-hand according to the individual’s residual EMG activity, with the advantage of making the system patient-specific and ensuring patients with even minimal EMG activity can be involved in the voluntary brain-stimulation protocol.

#### 2.2.3. Exoskeleton device

Electromyogram-triggered exoskeleton device was developed for rehabilitation of wrist-joint and fingers-joint (Singh et al., 2019a; Figure 1A) and used to move the affected-hand as-and-when intended in the brain-stimulation protocol. EDC muscle activity was chosen to trigger the exoskeleton device whenever the patient made the voluntary effort to extend the wrist. In the baseline position, the patient is instructed to put effort into wrist extension. The sequence of motion of the exoskeleton device was: wrist at the neutral position, finger-extension (baseline-position, Supplementary material) → wrist-extension and finger-flexion (final-position, Supplementary material) → wrist at neural position and finger-extension (back to the baseline-position). The range of Motion (ROM) of exoskeleton-device are customizable according to the comfort and clinical presentation (spasticity, contractures, and pain) of the patient, pertaining to one of the features of the exoskeleton device, hence, can accommodate a large patient population. The exoskeleton device is used for developing a platform for synchronizing TMS with the exoskeleton.

### 2.3. Participants

Patients were enrolled based on inclusion criteria: age 18–70 years, having ischemic/hemorrhagic stroke within 3–24 months, Mini-Mental Scale (MMS) = 24–30; Barthel-Index (BI) = 0–100, and Modified-Ashworth-Scale (MAS) = 1, 1 +, 2. MAS was used to measure spasticity at wrist-joint (Gregson et al., 1999; Takahashi et al., 2008; Sale et al., 2014; Singh et al., 2021). Three patients were enrolled, one patient each on MAS 1, MAS 1+, and MAS 2. Patients with contra-indication to TMS, no detectable Electromyogram (EMG) activity, and any other progressive neurological or cognitive disorders were excluded from the study. All patients signed the written informed consent. All patients were given care according to current clinical standards, as advised by the IRB. Three different persons executed three different aspects, applied the intervention, assessed the clinical data, and analyzed it; blinded to other’s observations with patients.

### 2.4. Data collection

All the participants underwent clinical assessment by a trained physiotherapists with more than 5 years of experience; a pre-therapy assessment a day before the initiation of the intervention sessions. The post-therapy assessment was performed a day after the completion of the intervention. The level of spasticity at the wrist joint measured by the MAS (MAS 0–4), the range of voluntary wrist movement defined in terms of the Passive Range of motion of the wrist (PROM 0–70 degrees) as measured by a goniometer, Barthel Index (0–100), and functional and sensorimotor-control of upper-limb as measured by Fugl-Meyer Scale Upper Limb (FMU/L 0–66) for whole arm, later segregated into its wrist hand (FMW/H) and shoulder elbow (FMS/E) components.

Cortical excitability was measured in terms of Resting Motor-Threshold (RMT) and Motor Evoked Potential (MEP) amplitude using Transcranial Magnetic Stimulation (TMS) over ipsilesional according to the standard protocol (Thair et al., 2017) on cortical representation area of EDC muscle between Cz and C3/C4 of the contralateral primary motor cortex with reference to the Electroencephalogram (EEG) cap. RMT was defined as the minimum intensity of TMS required to elicit an MEP in the target contralateral-muscle in five out of 10 trials, recorded in EMG, over the muscle cortical representation in the primary motor cortex. MEP obtained can be recorded as EMG activity in a target contralateral muscle. MEP encapsulates information relevant to the cortical excitability of the brain providing insights into membrane excitability of neurons, conduction and functional integrity of cortico-spinal tract, and neuromuscular junctions and is of prognostic importance in disease monitoring (Singh et al., 2019b). MEP should be ≥ 50 μv peak-to-peak amplitude at the hotspot in five out of 10 consecutive trials. The five MEP signals were then averaged for reporting.

### 2.5. Experimental set-up

The patient sat comfortably in the chair, kept forearm pronated, elbow joint at 90–120° flexion, wrist joint at a neutral position, and fingers at rest. The disposable gel-based wet Ag/AgCl surface electrodes were used in a bipolar configuration in which active electrodes were placed on the muscle belly of EDC with a center-to-center inter-electrode distance of 20 mm and the ground electrode was placed on the lateral epicondyle. Muscle contraction causing extension of the third digit of the hand was observed for identification of muscle belly and electrode placement. Electrodes were connected to the EMG amplifier and with TMS [type-D70 (AC), serial no. 0326, Magstim Rapid^2^, United Kingdom]. Specific hotspot for the EDC muscle was determined. Single-pulse TMS stimuli at the Resting motor threshold (RMT) were applied with the procedure widely used, using a flat 70 mm figure-of-eight coil placed tangentially with the handle pointing toward the back, 90° to the central sulcus and 45° to the midsagittal line for transsynaptic activation of the corticospinal tract (Rossini et al., 2015). TMS-stimuli were delivered by moving the coil in millimeters in all directions until the hotspot, producing maximum MEP response, was localized. Cortical-excitability once the hotspot was localized, RMT was measured by progressively increasing the Maximum Stimulator Output (MSO) starting from stimulus intensity of 35% in steps of 2–5% until a reliable MEP (>50 μV peak-to-peak) appears (Rothwell et al., 1999). Then, MSO is lowered in steps of 1% until there are five consecutive responses out of 10 trials. Each pulse was given at an interval between each stimulus of >5 s (Rossini et al., 2015).

### 2.6. Experimental protocol

Once the patient with spasticity is comfortable with the exoskeleton device mounted on the affected hand and can voluntarily make an effort for wrist extension, TMS-coil was set on the “hotspot” of EDC-muscle cortical-representation marked during RMT acquisition. Once the MEP was determined at the hotspot, the location of the hotspot was measured (with measuring tape) wrt nasion, inion, pre-auricular point, and Cz. The location of the hotspot was also noted on the 10–20 system EEG cap to maintain the position across the sessions. The stability of the hotspot of EDC muscle throughout the experiment was ensured by marking the area with a permanent marker (Werhahn et al., 1994; Awiszus, 2003; Thair et al., 2017; Singh et al., 2019b). The primary requirement of system hardware is to simultaneously trigger the TMS pulse and the exoskeleton-device, once the intention of the movement is detected in EMG. EMG-acquisition-system connected to the custom-designed hardware having a novel algorithm detects if the predefined activation threshold is crossed. Once the threshold is crossed, it simultaneously actuates the exoskeleton device and sends a Transistor-Transistor Logic (TTL) pulse to the TMS machine for generating a single-pulse TMS, in real-time.

The protocol had each motion trial fixed to 10 s that included a “voluntary-cue” for attempting the wrist extension (3 s), completing movement (5–7 s, depending on the motion parameters chosen based on clinical presentation), and remaining as the “rest-time” (remaining in 10-s; Figures 1B,C). During each 10-s trial, if (only) the voluntary effort is made within the voluntary-cue (3 s) and crosses the predefined EMG-activation-threshold, the controller in real-time simultaneously performs three tasks: (i) triggers single-pulse TMS at the hotspot, (ii) actuates exoskeleton-device (assisting wrist-extension), and (iii) provides performance-biofeedback (Figures 1B,C). A 10-s trial time was given to the patients with spasticity to put effort, completing the movement and relaxing the muscle with spasticity after the movement. 10 s is divided into three sections—3 + ~5 + ~1 s, hence, the first 3 s is a voluntary-trial to detect the EMG (to trigger TMS and Exoskeleton). Another 4–6 s to make movements assisted by the exoskeleton will depend on the clinical presentation of patients (range of motion, speed; Figure 1C). Since the protocol allows the experimenter to choose the motion parameters (range of motion and speed), depending on the patient’s clinical presentation (comfortable range of motion, contractures, and pain), each patient completed the movement at a different time, and the remaining few seconds (~1–2 s) will be given as a delay for getting the patient’s hand with spasticity relaxed and to maintain the consistency of protocol as 10 s trial. Depending on the individual clinical presentation, if the patient wants to take rest in between the trials due to fatigue, pain, or spasticity, the patient might make a voluntary effort after a gap of 1–2 s between any trials of 10 s each. If (only) the activation threshold is reached by voluntary effort, the controller triggers TMS-pulse and exoskeleton device and if it does not reach the predefined threshold in the first 3 s, trial-cycle is missed and the system is reset to begin a new trial, starting with visual voluntary-cue via green LED, maintaining the consistency of 10-s in the protocol. TMS-pulse is delivered within first-half of the EMG-burst generated by EDC (within the time of voluntary-trial time; Figure 1C) as soon as the predefined activation threshold on RMS-EMG is crossed, the average time-interval from the crossing of EMG-threshold to delivery of TMS-pulse being less than 50 ms.

As each complete trial (presented in Figure 1D) lasted 10 s (voluntary cue, movement, and rest time) and TMS stimulated the motor cortex once in 10 s by delivering single-pulse TMS, implying 0.1 Hz frequency, as used in literature (Buetefisch et al., 2011; Revill et al., 2020). Single-pulse TMS was applied at 100% Motor-Threshold, generating MEP at every pulse, synchronous with every voluntary wrist extension. If the patient with spasticity has to be voluntarily involved and with movement throughout the session, spasticity should be considered profoundly, i.e., time taken by muscle to initiate the movement, complete the movement, relax after the movement, and prepare for the movement the next trial.

Two types of bio-feedbacks in real-time were provided- intrinsic proprioceptive-biofeedback via exoskeleton-device assisting the movement and extrinsic adaptive visual-performance biofeedback, which was made proportional to RMS EMG amplitude. The visual feedback to the brain is given by the LEDs and the number of glowing LEDs is proportional to the effort by the patient during the assistance of movement (wrist extension) in real-time. Out of four EMG thresholds, other than the (first) activation threshold which triggered TMS-pulse and exoskeleton-device simultaneously, three other RMS EMG thresholds were pre-determined and calibrated as directly proportional to (glowing rows of LEDs) 8*8 dot-matrix and made adaptive after few trials. After the first 20 initial trials (as training), these three thresholds were used to make the EMG-performance biofeedback adaptive, (i.e., the number of times the thresholds were consistently attained/not attained), were used to constantly increase/decrease the visual performance feedback targets during device-motion in each cycle in real-time.

## 3. Results

All patients were able to comprehend and complete the session in time and tolerated sessions well with no complaints.

### 3.1. Demonstrating the activity-dependent TMS system in one session

After written informed consent, three patients (*n* = 3) were enrolled (Table 1) and clinical measures were obtained (Table 2). Each session of TMS synchronized with the exoskeleton-device and feedback (TSEF) platform, had ~270 trials of 10 s corresponding to 45 min and was given to three patients each. Three patients with different spasticity, on Modified-Ashworth-Scale (MAS = 1, 1+, and 2) at wrist-joint, completed their session in their timing (instead of 45-min session)- 50.85, 50.96, and 53.8 min respectively, with an average of 51.87 ± 1.67 min. The patients with (more) spasticity tend to take (more) inter-trial intervals and hence, with the increase in spasticity, the session time also increased. With respect to MAS-1 patients, patients with MAS-2 tend to take more time and had a difference of ~3 min (corresponding to ~18 trials of 10 s each) taken in between the trials. Additional 3 min’ rest were given to all the patients in between the session as rest.

**Table 1:** Details of enrolled patients.

Group	Patient	Sex	Age	Handedness	Stroke	Chronicity	Hypertension	Smoking and alcohol	Diabetic	Family history stroke	Mini mental scale
TSEF	P1	M	31	Right	R GC bleed	9	No	No	No	No	30
	P2	M	53	Right	L ACA, MCA infarct	3	No	No	No	No	29
	P3	M	25	Right	R MCA infarct	6	No	Yes	No	No	30
Physiotherapy control	P4	M	40	Right	R MCA infarct	8	No	No	No	Yes	30
	P5	M	38	Right	R MCA Lacunar infarct	9	No	Yes	Yes	No	30
	P6	M	42	Right	L Parietal & BG infarct	8	No	No	No	Yes	27

GC, gangliocapsular; ACA, anterior cerebral artery; MCA, middle cerebral artery; and BG, basal ganglia.

**Table 2:** Details of patients’ pre- and post-sessions measures of clinical scales and cortical-excitability.

Group	Patient	Chronicity	Age	MAS		BI		FMUE		FMWH		PROM		RMT		MEP	
				Pre	Post	Pre	Post	Pre	Post	Pre	Post	Pre	Post	Pre	Post	Pre	Post
TSEF	P1	9	31	2	1.5	75	90	34	40	8	12	15	30	100	78	0*	66.3
	P2	3	53	1.5	1	70	90	43	53	10	14	20	50	75	70	112	131
	P3	6	25	1	0	80	100	34	44	10	13	20	40	100	80	0*	60.5
	(Mean ± SD)	6 ± 3	36.3 ± 14.7	1.5 ± 0.5	0.8 ± 0.7	75 ± 5	93.3 ± 5.7	37 ± 5.2	45.6 ± 4.7	9.33 ± 1.15	13 ± 1	18.3 ± 2.8	40 ± 10	91.6 ± 14.4	76 ± 5.2	37.5 ± 64.9	86 ± 39.2
**Difference (Post-Pre)**				**0.67**		**18.3**		**8.6**		**2.6**		**21.7**		**15.6**		**48.5**	
**Relative % Improvement (Post-Pre)/Pre**				**46**		**24**		**23**		**31**		**118**		**18**		**129**	
Physiotherapy Control	P4	8	40	2	2	50	55	25	29	11	11	5	15	99	97	60	62
	P5	9	38	1.5	1.5	50	60	24	28	10	10	15	30	100	100	0	0
	P6	8	42	2	2	75	80	18	23	5	6	20	25	65	64	89	98
	(Mean ± SD)	8.3 ± 0.5	40 ± 0.2	1.8 ± 0.2	1.8 ± 0.2	58.3 ± 14.4	65 ± 13.2	22.3 ± 3.7	26.6 ± 3.2	8.6 ± 3.2	9 ± 2.6	13.3 ± 7.6	23.3 ± 7.6	88 ± 19.9	87 ± 19.9	49.6 ± 5.3	52 ± 49.2
**Difference (Post-Pre)**				**0**		**6.66**		**4.3**		**0.33**		**10**		**1**		**2.33**	
**Relative % Improvement (Post-Pre)/Pre**				**0**		**11.4**		**19.2**		**4.6**		**75**		**1.1**		**4.8**	

MAS (max 4): modified ashworth scale (a measure of spasticity).

BI (max 100): Barthel index (a measure of ADL).

FMUL (max 66): Fugl-Meyer upper limb.

FMWH (max 24): Fugl-Meyer wrist hand.

PROM (max 70): passive range of motion of wrist-joint.

RMT (%): resting motor threshold EDC hotspot.

MEP (μv): motor evoked potential amplitude.

∆ relative % clinical improvement = (Post clinical score—Pre clinical score/Pre clinical score)*100.

* MEP was not obtained even after giving 100% Maximum Stimulator Output due to decreased cortical-excitability after stroke (Chen et al., 2008).

Difference represents (Post Clinical score-Pre Clinical Score).

Bold are the derived parameters from values in Table 2 for easier understanding of readers.

### 3.2. Proof-of-concept study

After a demonstration of the TSEF protocol on three patients (section 3.1), a Proof-of-concept study was attempted for exploring the differences in clinical gains and cortical-excitability in patients with two groups: TSEF-group (*n* = 3, age = 36.3 ± 14.5, chronicity = 6 ± 3 months same patients as section 3.1) and physiotherapy group (*n* = 3, age = 40 ± 0.2, chronicity = 8.33 ± 0.5 months; serving as control). All six patients were right-handed, male with non-hypertension; five were non-diabetic, and two had a history of stroke in the family (Table 1). Clinical measures were acquired the day before the first-session and the day after the 20th-session (Table 2) along with the subjective-feedback (from TSEF-group, Table 3). In TSEF sessions, patients were motivated by the therapist for putting effort for wrist movement. TSEF sessions were given 45 min/per day for 20-sessions via the coil-holder system. The control-group received the dose-matched physiotherapy of 45 min/day for 20-sessions (details in Supplementary material).

**Table 3:** Patient’s Subjective Feedback given in the TSEF group.

S.No	Protocol	Patient’s feedback
1	Robotic Exoskeleton mounted on hand with the elbow at 90 degrees.	Difficult to maintain the elbow at 90 degrees with a TMS coil on the head.
2	Robotic exoskeleton on the flat-surfaced table.	The robotic exoskeleton’s table should be inclined such that the elbow could be 120 degrees to easily maintain the TMS coil on the head.
3	Table with the non-adjustable height of 30 in.	The height of the table on which the robotic exoskeleton is placed should be adjustable.
4	Thumb free during the wrist and finger joint movement.	A strap to hold the thumb should also be included in the exoskeleton.
5	Visual LED feedback placed on the table.	LED feedback should be in front of us to easily see without moving the eyes sideways as the head is fixed due to the TMS coil.
6	It was instructed to not use the proximal joints—Elbow, and shoulder and to put full effort into the wrist movement for EMG to be detected from EDC.	Stress on elbow and shoulder causing pain, a few minutes gap would be better.
7	The protocol had exoskeleton focusing only on wrist and fingers joint.	It should be made multi-joint to involve shoulder and elbow joints training too. It should not be limited to only the wrist and hand.
8	45 min of the session with additional 3-min of breaks in between the session.	I will not be able to do this for more than this duration.
9	The 10 s trial was made voluntary according to the patient. According to their clinical presentation (spasticity, pain, and contractures), they might/might not take a gap in between the trial and start the effort for wrist extension.	Initially, it was fine with six trials/min but after 5–10 min, it is easy if we have a 1–2 s gap between the trials.

Post 20-sessions, reduction in spasticity of wrist joint on MAS was also observed with one grade each in all three patients, however, control-group showed no change in spasticity post 20-sessions (Table 2). TSEF also showed considerable clinical gains (Barthel Index—mean 18.3, FMUE—mean 8.6 units, and PROM—mean 21.7 units), however, control-group showed minimal increase in clinical-gains (Barthel-Index—mean 6.6 units, FMUE—mean 4.3 units, and PROM—mean 10 units). FM for the upper-limb (FMUE) signifies measurement of sensorimotor functions of the whole arm including shoulder, elbow, wrist, and hand. Evaluating recovery at distal joints (Wrist/Hand) is critical in this study as it is focused on distal joints with exoskeleton training in TSEF sessions (along with stimulation of cortico-representation hotspot of EDC muscle responsible for wrist extension movement). Hence, we segregated FMUE (FM-upper-limb) into proximal-joints FMSE (FM-Shoulder/Elbow) and distal-joints FMWH (FM-Wrist/Hand). Post 20-sessions, in TSEF-group FMWH score was observed to increase in all three patients (P1 by 4 units, P2 by 4 units, and P3 by 3 units), however, control-group showed improvement in only one patient by 1 unit (Table 2).

Pre-therapy, MEP were not obtained in two patients (P1 and P3) in TSEF-group and one patient (P5) in control-group, even after giving 100% Maximum Stimulator Output possibly due to decreased cortical-excitability after stroke (Chen et al., 2008) and for these patients, hotspot was determined for the unaffected hand at unaffected-hemisphere and the corresponding measurements were made for the affected-hemisphere, a standard procedure described in the literature (Chang et al., 2010; de Freitas et al., 2023). After 20 intervention-sessions in TSEF-group, patients demonstrated improvement in cortical-excitability (Table 2) i.e., P1 and P3 showed the appearance of MEP at decreased RMT. The mean MEP increased by ~48.5 μV at a decrease of mean RMT by ~15.6%, however, post 20 physiotherapy-sessions in control-group, the cortical-excitability remained same for all the patients with mean MEP increased by ~2.33 μV at a decrease of mean RMT by ~1% (Table 2).

Subjective feedback (Table 3) by patients showed the experience of TSEF sessions WRT patients’ experience. Adjustments in the table’s height and angles and arrangement for the thumb were suggested along with a request to make it a multi-joint training device. The patient suggested that due to spasticity, the duration of use could not be longer due to fatigue caused in the hand.

## 4. Discussion

A novel Activity-Dependent TMS with a two-way feedback system and novel protocol, to voluntarily involve the patient throughout the sessions, has been developed and its functioning has been demonstrated along with a feasibility-study (intervention *n* = 3 and controls *n* = 3). We attempted to close the loop between the intrinsic brain state, cortical stimulation, and biofeedback (intrinsic-proprioceptive and extrinsic-visual) to the brain. In every 10-s trial, the EMG-triggered TMS platform uses real-time EDC-muscle EMG to simultaneously (a) trigger impaired EDC muscle cortical representation (by voluntary-activated EDC muscle activity); (b) actuate exoskeleton-device utilizing use-dependent-plasticity and providing intrinsic-proprioceptive-biofeedback via skin-mechanoreceptors, muscle-spindles, joints, etc., by assisting the attempted movement completing the sensorimotor-loop (Gomez-Rodriguez et al., 2011); and (c) provide extrinsic adaptive visual performance-biofeedback, considering the criticality of intrinsic and extrinsic-biofeedback in post-stroke recovery (Gomez-Rodriguez et al., 2011). This strategy potentially synchronizes a group of neurons excited for muscle activity and exoskeleton training with another group of neurons through TMS-pulse leading to the strengthening of synaptic connections between these two groups of neurons, facilitating Hebbian-learning (Gerstner, 2011; Revill et al., 2020). Moreover, neuroplasticity happens in synaptic connections between motor neurons firing voluntarily and neurons activated by stimulation (Edwardson et al., 2013). In non-human primates, motor-cortex activity associated with movement lasts for 250 ms after EMG-onset, therefore, TMS-pulse was applied to the motor cortex within the first half of EMG-bursts, to arrive synchronously with cortical activity in the motor cortex generating wrist-extension (Revill et al., 2020).

Using a functionally important and often affected muscle, i.e., EDC is neuro-anatomically and physiologically justified and was attempted to target impairment-oriented-functional-neuroplasticity, unlike the APB muscle used commonly (Izumi et al., 2008; Hoogendam et al., 2010). Considering the criticality of feedback in stroke rehabilitation, the proprioceptive feedback during exoskeleton assistance is considered an overarching somatosensory-theme, through mechanoreceptors consisting of joint-position sense, kinaesthesia, sense of force, and sense of joint-velocity on stretch-receptor in ligaments and muscle-spindles (Ager et al., 2020). The proprioceptive feedback to the brain is known to modulate the ongoing cortical activity, completing the sensorimotor loop (Darvishi et al., 2017), which we are exploring in this study (during the assistance of wrist extension by exoskeleton-device simultaneous with the MEP at every trial). Post-sessions, the relative % change in all clinical scores was observed in both groups. With TSEF-group, MAS showed a decrease of 46%, with an increased Barthel Index of 24%, FMUE 23%, and PROM 118%, however, in control-group, MAS does not show any change, with increased Barthel Index of 11%, FMUE 19%, and PROM 75%. It is worth noting that in TSEF-group, FMWH, a relevant joint in training, showed a considerable increase of 31% compared to only ~5% increase in control-group (Table 2).

The improvements in cortical-excitability (of EDC-hotspot involved in cortical stimulation) were observed in TSEF-group. Post-sessions, 2/3 of patients showed the appearance of MEP at decreased RMT. In TSEF-group, MEP was increased by 129% at a decrease of RMT by 18%, however, in control-group, the MEP was observed to increase by only ~5% at a decreased RMT of ~1% (Table 2). Increased cortical-excitability with decreased spasticity, along with specifically increased FMWH and passive-ROM indicated functional gains. Patients seemed more confident in ADLs like holding the door knob or having a glass of water while continuously holding the glass. As the functional gain is critically dependent on the motor cortex, improved cortical-excitability (increased MEP at decreased RMT), and clinical gains observed in this might suggest clinically relevant neuroplasticity (Revill et al., 2020). Although, improvement might be pertaining to exoskeleton training alone and needs to be further investigated in the future with larger-cohort, the clinical gains and increase in cortical-excitability are in line with the ADS studies in literature for animals, healthy and stroke patients, indicating the feasibility of patient-specific ADS with feedback to capitalize the unique physiology resulting in robust neural plasticity (Edwardson et al., 2013; Revill et al., 2020). The subjective feedback asserted towards the acceptability of the protocol and precious suggestions and feedback, such as placement of visual feedback, the height of the table where the exoskeleton is placed, about improving the comfort for future protocols and their acceptance and “look forward” approach towards such protocols for other proximal joints as well.

Spasticity is a rarely discussed obstacle in stroke-rehabilitation literature, which might be pertaining to the clinical challenges involved in dealing with spasticity. TSEF sessions showed to decrease the spasticity in each patient, and have the potential to be tailored to each patient with different spasticity. The critical effect of spasticity from MAS 1 to 1+ to 2 was observed in this feasibility-study for the TSEF-group, the inter-trial interval was found to be increased with an increase in spasticity, indicating the importance of individualized patient-specific tailored-protocol with inter-trial interval and “rest-time” within each trial. A similar observation is also indicated by patients’ subjective-feedback number-9 (Table 3) which speaks profoundly about the acceptability of the individualized protocol tailored according to the spasticity as the patient can decide the inter-trial interval. If a patient with spasticity has to be involved in a therapy process that is meant to be voluntary and with the movement, therapy should take the spasticity as a critical consideration, i.e., time taken by the muscle to initiate the movement, complete the movement, relax after the movement, and prepare for the movement for the next trial. The rationale behind 0.1 Hz frequency TMS for the proposed customized Hebbian stimulation was that the patient (with spasticity) has to be voluntarily involved throughout the therapy process and cortical stimulation should be synchronized with neural activity (attempt of wrist extension).

The proposed protocol was voluntary-activated with patient-specific inter-trial intervals, to ensure the patient with spasticity makes effort only when comfortable enough, patient was actively and voluntarily involved in the process instead of passively sitting during rTMS-therapy. With the given advantage of the exoskeleton being customizable in terms of motion parameters according to different ranges of clinical presentation (spasticity, contractures, and pain), finger height-support, voluntary-activated, and configurability of residual-EMG of individual-patient, the individualized and voluntary-activated protocol might be able to serve large patient-population. The main limitation of this proof-of-concept study is the small number of patients and the absence of long-term outcome assessments. Since, all the patient cohort included in this study were found to be young, generalization of results to a larger cohort of the patient population is not possible and warrants further investigation on a larger cohort.

## 5. Conclusion

An individualized TSEF platform having the potential to be tailored to each patient with different spasticity has been developed, and demonstrated and its clinical potential has been evaluated in the feasibility-study. An increase in cortical-excitability and clinical-gains were observed which was not observed in control-group. Being voluntarily involved during the whole brain stimulation protocol might add clinically relevant neuroplasticity.

## Data availability statement

The original contributions presented in the study are included in the article/Supplementary material, further inquiries can be directed to the corresponding author.

## Ethics statement

The studies involving human participants were reviewed and approved by Institute Review Board, All India Institute of Medical Sciences, Delhi, India. The patients/participants provided their written informed consent to participate in this study.

## Author contributions

NS: conceptualization, methodology, data curation, writing—original draft, writing—review and editing. MS: data curation, intellectual feedback about patients, writing—review and editing. NK and MP: writing—review and editing, scientific resources, and supervision. AM: conceptualization, methodology, supervision, scientific resources, and writing— review and editing. All authors contributed to the article and approved the submitted version.

## Funding

This work was supported by SERB-DST, India (YSS/2015/000697) and Abdul Kalam Technology Innovation National Fellowship by Indian National Academy of Engineering (INAE/121/AKF/38).

## Conflict of interest

The authors declare that the research was conducted in the absence of any commercial or financial relationships that could be construed as a potential conflict of interest.

## Publisher’s note

All claims expressed in this article are solely those of the authors and do not necessarily represent those of their affiliated organizations, or those of the publisher, the editors and the reviewers. Any product that may be evaluated in this article, or claim that may be made by its manufacturer, is not guaranteed or endorsed by the publisher.
