# Supplementary material for: Individualized closed-loop TMS synchronized with exoskeleton for modulation of cortical-excitability in patients with stroke: a proof-of-concept study
Source: Front Neurosci. 2023 May 25;17:1116273. doi: 10.3389/fnins.2023.1116273 (PMC10248009; doi:10.3389/fnins.2023.1116273)
Supplement: Supplementary file 1 [file Data_Sheet_1.PDF]

Individualized closed-loop TMS synchronized with Exoskeleton for modulation of cortical-excitability in patients with Stroke: A Proof-of-Concept Study

Supplementary material

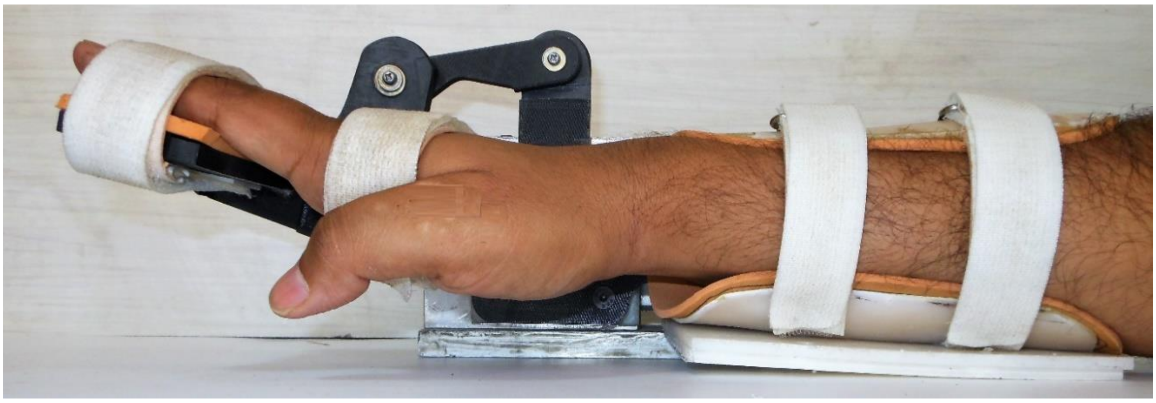

Supplementary Figure 1a: Exoskeleton device in baseline position- (wrist in neutral position with fingers in extension position)

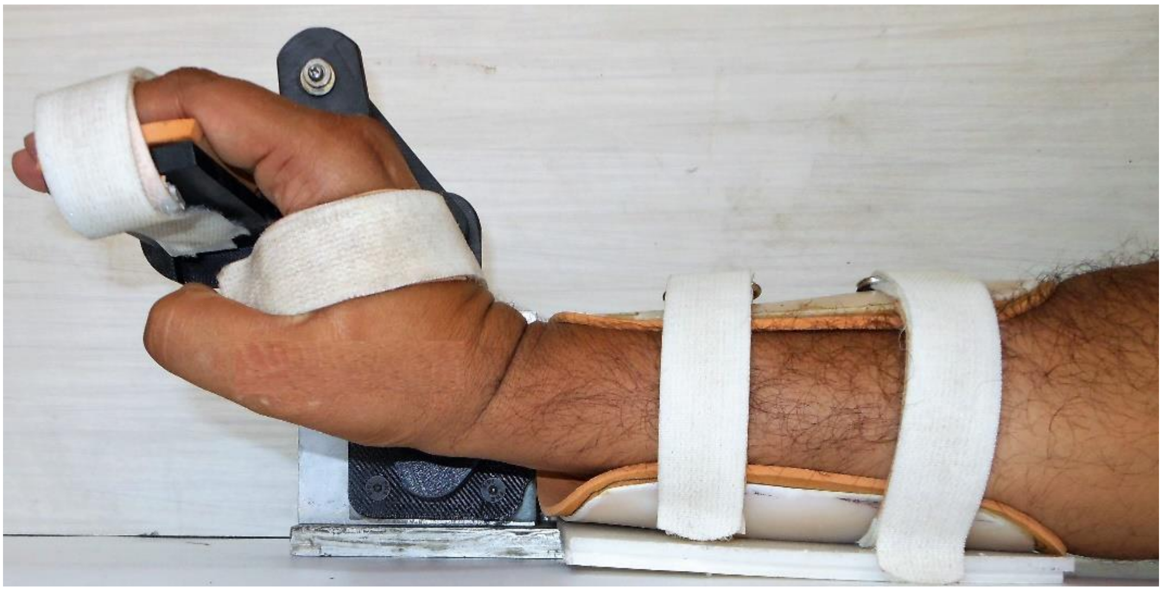

Supplementary Figure 1b: Exoskeleton device in final position (wrist in extension position with fingers in flexion)

(This figure reprinted by permission from IEEE Trans Neural Syst Rehabil Eng, from an article by Singh N et al.) Singh N, Saini M, Anand S, Kumar N, Srivastava MVP, Mehndiratta A. Robotic Exoskeleton for Wrist and Fingers Joint in Post-Stroke Neuro-Rehabilitation for Low-Resource Settings. IEEE Trans Neural Syst Rehabil Eng [Internet]. 2019 [cited 2019 Sep 27];1–1. Available from: https://ieeexplore.ieee.org/document/8846101/

Details of ‘Conventional therapy-sessions’:

- In our cohort, stroke patients exhibited impairment in upper extremity paresis. Consistent with prior research, the degree of recovery in the paretic hand and fingers was found to substantially influence patients' perception of recovery from this condition. Accordingly, motor relearning exercises concentrating on deficits in gross and fine movements of the wrist and hand were employed to address limitations in patients' active functional participation in basic and instrumental activities of daily living.
- The control group underwent a training session that lasted 45 minutes, 5 days per week, for a duration of 4 weeks, which was identical to the intervention group. The activity type, intensity, and frequency were individualized according to the patient's baseline clinical presentation, as measured by clinical scales such as the Modified Ashworth Scale (MAS), Fugl-Meyer Assessment (FMA), Barthel Index (BI), Brunnstrom scale, and Range of Motion (ROM).
- Tasks specific to upper extremity included:
    - Passive stretching of long wrist flexors with the hold of 30 sec followed by relaxation
    - Fist making in prone and thrust release-10 reps (3sets)
    - Fist making in mid prone position -10 reps (3sets)
    - Ball squeezing and release-10 reps
    - Lock and key movements -10 reps
    - Muscle facilitation techniques like stroking, brushing, icing - 3-5 strokes twice in a day
    - Task-oriented training (TOT); reaching, grasping a tool, lifting a glass of water, pen holding, bottle opening.

- The task difficulty was systematically increased in response to improvement in upper extremity activities such as reaching, grasping, lifting, and steady holding, which required an optimal range of motion of the shoulder, elbow, wrist, and hand, as well as smooth inter-joint coordination and optimal task performance speed, compared to the unaffected extremity of the individual patient.
- The task was made challenging by adding gradations like:
    - Gross to fine movements
    - Static to dynamic positioning
    - Single to dual activity
    - Non weighted to the incorporation of weights
    - Indoor to the outdoor environment
- If the patient reported any pain, fatigue, or difficulties with balance or coordination during the execution of a specific task involving the wrist/hand or proximal joints such as the shoulder/elbow, the task was fragmented into smaller segments for ease of practice. The task was then gradually progressed towards achieving the required functional goal.
